# Nonsurgical endodontic management of maxillary sinusitis of endodontic origin: a CBCT-guided case report

**DOI:** 10.3389/froh.2026.1901082

**Published:** 2026-07-30

**Authors:** Mai Alzarooni, Abdul Rahman Saleh, Firas Elmsmari, Abayomi Omokeji Baruwa

**Affiliations:** 1 Department of Clinical Sciences, College of Dentistry, Ajman University, Ajman, United Arab Emirates; 2 Center of Medical and Bio-allied Health Sciences Research, Ajman University, Ajman, United Arab Emirates

**Keywords:** apical periodontitis, cone beam computed tomography, endodontics, maxillary sinusitis of endodontic origin, sinusitis

## Abstract

**Background:**

Maxillary sinusitis of endodontic origin (MSEO) is a recognized complication of odontogenic infection, arising from the close anatomical relationship between the posterior maxillary dentition and the maxillary sinus floor. Failure to identify the odontogenic etiology of sinus pathology risks misdiagnosis, delayed treatment, and unnecessary surgical intervention. This case report presents the diagnostic approach and clinical management of a maxillary second molar associated with sinus mucosal thickening, emphasizing the role of advanced imaging and conservative endodontic therapy in achieving successful resolution.

**Case description:**

A 38 year old female patient was referred for completion of previously initiated root canal treatment on tooth #27. Clinical examination and review of presenting symptoms including a year-long increase in headache frequency and intermittent rhinorrhea in the absence of typical upper respiratory signs were supplemented with cone beam computed tomography (CBCT). Imaging revealed thickening of the Schneiderian membrane and close proximity of the palatal root apex to the maxillary sinus floor, establishing an endodontic etiology for the sinus pathology. Nonsurgical root canal treatment was completed following adequate chemomechanical preparation and obturation techniques, with mineral trioxide aggregate (MTA) placed in the palatal canal. One-year clinical and radiographic follow-up demonstrated a significant reduction in sinus mucosal thickening and complete resolution of symptoms.

**Conclusion:**

This case report highlights the importance of recognizing MSEO as a distinct clinical entity requiring accurate etiological diagnosis. It further demonstrates that CBCT imaging is essential in identifying sinus involvement of odontogenic origin, and that conservative nonsurgical endodontic therapy can effectively resolve associated sinus pathology, thereby preventing unnecessary surgical intervention and improving patient outcomes.

## 1 Introduction

An undiagnosed maxillary sinusitis of odontogenic etiology leads to surgical overtreatment. Periapical infection is a well-recognized cause of maxillary sinusitis and the successful management of apical periodontitis through endodontic therapy represents an effective and conservative approach to resolving associated maxillary sinusitis of endodontic origin (1). The maxillary sinuses are pneumatized cavities situated within the maxillary bones, interposed between the orbital and nasal cavities, and lined by a thin mucosal layer known as the Schneiderian membrane, composed of a pseudostratified ciliated columnar epithelium (2, 3). These sinuses may extend toward the alveolar process of the maxillary bones, establishing a close anatomical relationship with the root apices of the posterior maxillary dentition, most notably the molars and second premolars (4). As a consequence of this proximity, periapical lesions of the maxillary posterior teeth can give rise to odontogenic infection involving the maxillary sinus (5, 6). Established sources of odontogenic infection contributing to this process include pulp necrosis, periodontal bone loss, oroantral fistula, and foreign materials introduced during dental procedures (7, 8). Maxillary sinusitis of endodontic origin (MSEO) may consequently manifest as periapical osteoperiostitis (PAO), characterized by a distinctive “halo” lesion of the maxillary sinus periosteum, or as periapical mucositis (PAM), in which infectious agents of apical origin stimulate reactive antral mucosal proliferation (9).

Although the etiology of odontogenic sinusitis is well established in both clinical and scientific literature, challenges persist in achieving accurate diagnosis and appropriate management from both dental and otolaryngological perspectives (5, 10, 11). MSEO is estimated to account for up to 72% of all cases of unilateral maxillary sinusitis, yet referrals for endodontic evaluation from otolaryngologists and maxillofacial surgeons remain infrequent (10, 12, 13). This gap in interdisciplinary referral persists despite mounting evidence that MSEO can be successfully resolved through nonsurgical endodontic therapy alone (14), with conservative endodontic management remaining underutilized relative to more invasive surgical approaches (11).

Cone beam computed tomography (CBCT) has been increasingly integrated into contemporary dental practice, enhancing diagnostic accuracy, treatment planning, and follow-up assessment. By facilitating informed clinical decision-making and enabling more accurate prediction of treatment outcomes, CBCT has proven to be of considerable benefit to both patient care and clinical practice (15, 16). This case report therefore aims to demonstrate the successful nonsurgical management of MSEO through CBCT-guided diagnosis and the application of contemporary endodontic techniques.

## 2 Clinical procedures

### 2.1 History, clinical examination and general procedures

This case report has been written according to CARE reporting guideline (17). A 38-year-old female patient presented to the postgraduate endodontic clinic for completion of a previously initiated root canal treatment on tooth #27. The treatment had been initiated two weeks earlier by a general dental practitioner in a private practice setting. The patient was systemically healthy (ASA Class 1), reported no current medication use, and had no known drug allergies. The tooth had a history of repeated fractures of a composite resin restoration over the preceding year. At the time of the most recent dental visit, the tooth was diagnosed as non-vital and endodontic treatment was initiated. Upon further questioning, the patient reported an increased frequency of headaches over the past year, accompanied by intermittent rhinorrhea occurring in the absence of any symptoms suggestive of an upper respiratory tract infection. Extraoral examination revealed no facial swelling or asymmetry. Intraoral assessment demonstrated satisfactory oral hygiene, with both soft and hard tissues within normal limits. No swelling, sinus tract, or tenderness to percussion was detected. The initial periapical radiographic assessment reportedly showed no evidence of periapical pathosis. The case was therefore classified as a previously initiated root canal treatment with no radiographic evidence of apical pathology.

### 2.2 First clinical visit

After obtaining informed consent, local anesthesia was achieved using 2% lidocaine with 1:80,000 epinephrine administered via buccal infiltration, and the tooth was isolated using a rubber dam. The temporary restoration was removed, a composite resin build-up was placed to reconstruct the mesial wall and improve isolation, and the access cavity was refined under magnification (CJ-Optik, Wetzlar, Germany). Only three canals, palatal (P), mesiobuccal (MB), and distobuccal (DB) were identified and negotiated. Drying of the palatal canal was challenging, and the presence of clear exudate was noted on paper points. The canals were dressed in a non-setting calcium hydroxide intracanal medicament, sealed with a glass ionomer temporary restoration, and the patient was referred for CBCT imaging. On assessing the CBCT scan, there was a significant thickening of the Schneiderian membrane of the left maxillary sinus in comparison with the contralateral side, with a maximum mucosal thickness of approximately 6.6 mm. The palatal root apex appeared to be in close proximity to, or penetrating, the maxillary sinus floor, and slight apical root resorption was observed (Figure 1).

**Figure 1:**
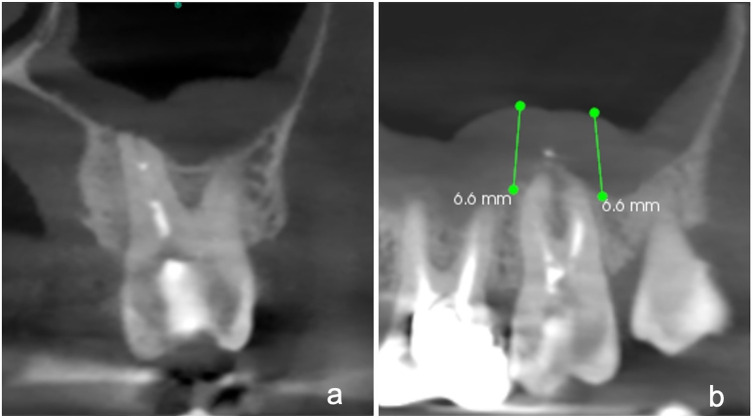
Preoperative radiographic and CBCT assessment of tooth #27. **(a)** Coronal and **(b)** sagittal CBCT views demonstrating close proximity of the root apices to the maxillary sinus floor and associated thickening of the sinus mucosal lining.

After the visit, the clinical findings and previous history were correlated. Differential diagnosis of non-odontogenic and odontogenic causes was considered. Non-odontogenic etiologies, including allergic rhinitis, chronic rhinosinusitis, and sinonasal neoplasia were considered less likely due to the absence of nasal obstruction and anosmia. Other possible odontogenic sources including periodontal disease, oroantral communication from the adjacent teeth and foreign materials were also considered however the intraoral and radiographic examination identified none of these. The final diagnosis of maxillary sinusitis of endodontic origin relating to tooth #27 was established based on confirmed incomplete prior endodontic treatment, clear exudate from the palatal canal, and CBCT evidence of localized, unilateral Schneiderian membrane thickening with root in close proximity to the sinus.

### 2.3 Second clinical visit

At the subsequent appointment, working length determination was performed using an electronic apex locator in conjunction with intraoral periapical radiography. Gauging of the palatal canal was achieved using a size 55K-file. Biomechanical preparation was performed using a nickel–titanium rotary system WaveOne Gold (Dentsply Maillefer, Ballaigues, Switzerland) Glider, Small, and Primary files in a sequential protocol for the MB and DB canals, while a circumferential brushing motion was applied in preparing the palatal canal to improve debridement of the canal walls. The final irrigation protocol consisted of 2% sodium hypochlorite (NaOCl) activated with an ultrasonic device (IriSafe tip) for three cycles, followed by 5 mL of 17% ethylenediaminetetraacetic acid for one minute to facilitate smear layer removal, and a final rinse with 2% NaOCl. In view of the close anatomical relationship between the palatal root apex and the maxillary sinus floor, the palatal canal was obturated using mineral trioxide aggregate (MTA; ProRoot MTA, Dentsply Sirona, USA) delivered using an MTA carrier, with the remaining canal space backfilled using thermoplasticized gutta-percha. The MB and DB canals were obturated using calibrated gutta-percha and a resin-based sealer (AH Plus, Dentsply Sirona, Konstanz, Germany) with the continuous-wave of condensation technique. A definitive composite resin restoration was placed, and the patient was scheduled for full-coverage crown placement and a follow-up visit was arranged (Figure 2).

**Figure 2:**
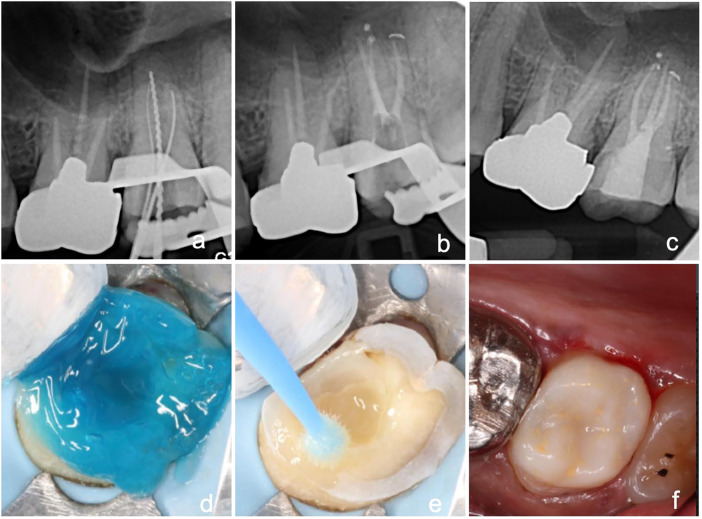
Intraoperative and postoperative documentation of root canal treatment. **(a)** Periapical radiograph confirming working length determination. **(b)** Periapical radiograph verifying the quality of root canal obturation. **(c)** Postoperative periapical radiograph following placement of the composite restoration. **(d–f)** Sequential clinical photographs illustrating the stages of adhesive preparation, including etching, bonding, and final crown cementation.

### 2.4 Follow-up evaluation

The patient was followed up through telephone consultations during the first few weeks after treatment and at regular intervals up to three months to monitor symptom resolution. At the one-year recall, she reported a marked reduction in the frequency of headache episodes and complete resolution of the intermittent rhinorrhoea. She explained that these symptoms had been present for approximately one year before treatment and that she had never associated them with the affected tooth. Following completion of the root canal treatment, she experienced complete relief from both the headaches and nasal symptoms, expressing significant satisfaction with the outcome. These clinical improvements were corroborated by follow-up periapical radiographs and CBCT imaging, which demonstrated a substantial reduction in the maxillary sinus mucosal thickening on the affected side, with the mucosal thickness approaching normal limits and becoming comparable to that of the contralateral sinus (Figure 3). There was no recurrence reported or any other complications relating to the treatment.

**Figure 3:**
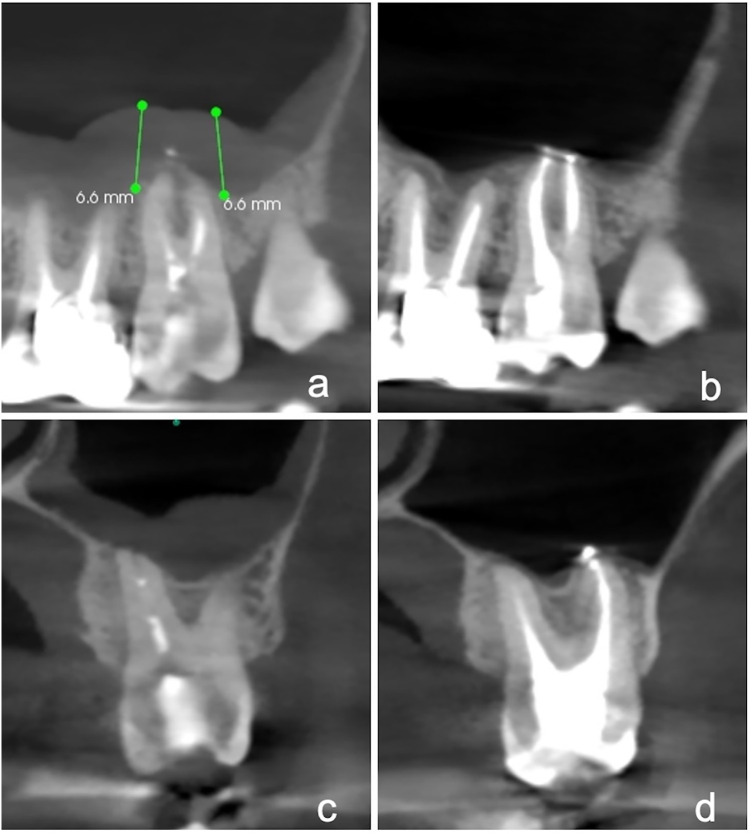
Coronal and sagittal CBCT views at baseline and one-year follow-up. **(a)** Preoperative and **(b)** postoperative coronal CBCT views demonstrating thickening of the maxillary sinus mucosal lining at initial presentation and its substantial reduction at the one-year recall, respectively. **(c)** Preoperative and **(d)** postoperative sagittal CBCT views confirming the same pattern of mucosal resolution on the affected side.

## 3 Discussion

The American Association of Endodontists (AAE) introduced the term maxillary sinusitis of endodontic origin to specifically categorize sinusitis resulting from endodontic infection (10). This is distinct from the broader classification of maxillary sinusitis of odontogenic origin (MSOO), which encompasses sinusitis arising from any adjacent dental pathology or iatrogenic injury sustained during dental procedures, including apical lesions caused by pulp necrosis, periodontal bone loss, oroantral fistula, or foreign materials introduced during dental treatment (3, 7, 18–20). Evidence from the literature indicates that periapical lesions of endodontic origin can spread into the maxillary sinus, with maxillary molars being eleven times more likely than premolars to induce MSEO owing to their closer proximity to the sinus floor (13, 14). The close proximity or direct continuity between the periradicular region of the maxillary posterior teeth and the sinus floor permits periapical lesions to involve these pneumatized anatomical spaces. Bacterial dissemination may occur via direct diffusion through the porous maxillary bone or indirectly through lymphatic and vascular channels, resulting in localized or generalized mucosal changes within the sinus (6).

Odontogenic infection may gain access to the sinus through rupture of its floor, direct diffusion through the porous maxillary bone, or indirectly via lymphatic and vascular channels (21). Progressive bone loss associated with periapical lesion development further facilitates bacterial infiltration and proliferation within the sinus mucosa, resulting in mucosal thickening that is identifiable on radiographic examination. Despite being a subject of repeated investigation, no consensus exists regarding the prevalence of MSOO, with reports ranging between 10% and 93% across primary studies (8, 20). Accurate identification of the odontogenic etiology is central to treatment efficacy, as failure to do so is likely to result in therapeutic failure, recurrent episodes, and potential progression to more severe manifestations (19, 22). This diagnostic challenge is compounded by the fact that the implicated dental lesions are frequently asymptomatic and do not present as an immediately evident cause of sinusitis, even when occasionally symptomatic or responsive to percussion (23). Vitali et al. (24) further demonstrated that apical lesions, periodontitis, and moderate-to-severe bone loss are significant predictors of odontogenic sinusitis, with severe bone loss increasing the risk nearly fourteen-fold, underscoring the importance of a thorough dental evaluation in all cases of unilateral maxillary sinusitis.

The clinical presentation of MSOO is often non-specific and may progress at a variable rate. Thermal sensitivity is rarely a feature, as the source teeth in MSEO are typically necrotic or have undergone failed endodontic treatment. Similarly, periapical pain is usually absent, given that infection decompresses into the sinus through a fistula, thereby relieving local pressure; edema and intraoral sinus tracts are consequently uncommon. As with many endodontic diagnoses, etiology cannot be determined by radiographic assessment alone, and a thorough clinical evaluation of pulpal status is required to differentiate periapical mucositis from other mucosal pathologies. Radiological imaging therefore plays a critical role in identifying the etiological basis of the condition and guiding subsequent management (10, 25).

Accurate diagnosis of odontogenic maxillary sinusitis requires a comprehensive understanding of the dynamic interrelationship between the tooth, bone, and maxillary sinus complex (26, 27). While periapical radiographs remain the most widely employed imaging modality in endodontics, conventional two-dimensional imaging presents considerable interpretive limitations in the posterior maxilla (28). CBCT is therefore recommended for the diagnosis of MSEO, as it permits comprehensive three-dimensional evaluation of the relevant anatomical structures and their interrelationships, and this capability directly informed its application in the present case (27). Radiographic features of sinus pathosis may manifest as sinus opacity, mucosal basal thickening, sinus polyp, or antral pseudocyst, with mucosal basal thickening representing the most prevalent finding, reported in up to 90% of cases (29, 30).

Root canal treatment is the primary intervention indicated for the resolution of MSEO, and precise diagnosis necessitates a comprehensive assessment of the patient's medical and dental history in conjunction with a thorough clinical examination and interpretation of imaging findings (27, 31). As illustrated in the present report, this systematic diagnostic approach was central to the accurate identification of the odontogenic etiology and ultimately facilitated successful management. Following elimination of the primary infection, clinical and radiographic review should be conducted at appropriate intervals to confirm regression of the associated sinus pathology, which generally occurs within a few months of treatment (26, 32, 33). In the present case, periapical radiographs and CBCT obtained at the one-year recall showed a substantial reduction in maxillary sinus mucosal thickness, consistent with the resolution timeframe reported in the literature.

Regarding adjunctive therapy, systemic antibiotic use in MSEO should adhere strictly to clinical guidelines, prescribing exclusively as an adjunct in cases of systemic involvement or rapidly spreading infection, rather than as a primary or definitive treatment modality. Clinical management must prioritize endodontic disinfection, with surgical intervention reserved for persistent cases or severe acute presentations requiring immediate drainage (34). In accordance with current guidelines, optimal outcomes in MSEO are best achieved through close interdisciplinary collaboration, with both endodontists and otorhinolaryngologists contributing to treatment decision-making within their respective domains of competence. The literature consistently emphasizes the importance of well-defined referral pathways between these specialties, as timely and coordinated management remains fundamental to optimizing clinical outcomes for patients with this condition (10, 32, 35).

## 4 Conclusion

This report highlights the importance of advanced imaging in the diagnosis and conservative endodontic management in resolving maxillary sinusitis of endodontic origin. The CBCT was instrumental in delineating the anatomical relationship between the tooth and the maxillary sinus, enabling etiological diagnosis and informed treatment planning without the need for surgical intervention.

## Data Availability

The original contributions presented in the study are included in the article/Supplementary Material, further inquiries can be directed to the corresponding authors.
